# Re-admissions to hospital and patient satisfaction among patients with chronic obstructive pulmonary disease after telemedicine video consultation - a retrospective pilot study

**DOI:** 10.1186/2049-6958-9-6

**Published:** 2014-01-30

**Authors:** Safaa Saleh, Jan Petter Larsen, Johannes Bergsåker-Aspøy, Heidi Grundt

**Affiliations:** 1 Stavanger University Hospital, 4011 Stavanger, Norway; 2 Norwegian Centre for Movement Disorders, Stavanger University Hospital, Stavanger, Norway; 3 University of Bergen, Bergen, Norway

**Keywords:** Chronic obstructive pulmonary disease (COPD), Hospital re-admission, Length of hospital stay, Patient satisfaction, Telemedicine video consultation (TVC)

## Abstract

**Background:**

Chronic obstructive pulmonary disease (COPD) is a major cause of acute hospital admissions. The main object of our study was to evaluate the effects of telemedicine video-consultation (TVC) on the frequency of hospital re-admissions due to COPD exacerbations. Our secondary aim was to assess the impact of TVC on the length of re-admission stays within 6 and 12 months follow up after TVC. Patient satisfaction was also evaluated.

**Methods:**

The study was a retrospective observational study of COPD patients who after hospital discharge or during outpatient treatment for acute COPD exacerbations, were monitored for 2 weeks by TVC at home by a specialist nurse at the hospital during a pilot project period. Retrospectively, we compared the frequencies (chi-square test) and durations of hospital re-admissions (paired t-test) due to COPD exacerbations within 6 and 12 months follow up after TVC to comparable events 6 and 12 months prior to TVC.

**Results:**

Among 99 patients followed for 6 months after TVC, 56 were followed for totally 12 months. The number of patients re-admitted and the number of re-admissions due to COPD exacerbations were not reduced within 6 or 12 months post-TVC, as compared to 6 and 12 months pre-TVC.

The mean length of re-admission stays within 12 months post-TVC was markedly reduced as compared to pre-TVC. Patients hospitalised the last 6 and 12 months pre-TVC, had significantly shorter re-admission stays, p = 0.033 and p = 0.001, respectively. Patient satisfaction was high.

**Conclusion:**

Despite the failure to demonstrate reduced frequency of re-admissions within 6 and 12 months post-TVC, the re-admission length within 12 months post-TVC was markedly reduced as compared to pre-TVC. The patient satisfaction was high. Future prospective, randomised, controlled trials must be performed before TVC can be recommended in COPD management.

## Background

Chronic obstructive pulmonary disease (COPD) is a major cause of acute hospital admissions [1-3]. Approximately 15,000 acute hospital admissions per year in Norway [4], 20% of all emergency admissions, are due to acute COPD exacerbations. In Norway, 20,000 COPD patients have severe disease with forced expiratory volume in the first second (FEV_1_) < 50% of expected value [3]. As the severity of the disease increases, exacerbations are more frequent, and the hospitalisation frequency increases [5]. In the study by Eagen et al. [4], 13% of patients in Western Norway admitted with an acute COPD exacerbation were re-admitted within the first 30 days after discharge, in accordance with the findings from a Danish study [6]. The rate of re-admission within one year in the Danish study was 46% [6], while British reports have suggested that 30% were re-admitted within 90 days [7].

Several reports published during recent years have studied if COPD care can be improved through nurse telemedicine consultation when monitoring COPD patients at home and thus if it can prevent hospital admissions [8-16]. However, few studies implement telemedicine video-consultation (TVC) when monitoring COPD patients at home [12,16,17], using information technology to monitor patients at home, while the clinician stays in the hospital.

Recent experiences from a non-randomised study, using TVC in Denmark when monitoring COPD patient in their homes [17], have shown 14% reduction of early re-admissions (within 28 days), probably related to early onset of treatment of exacerbations, with rapid effect and less drug use.

The Dalane District Medical Centre (DMC)/Egersund Hospital, part of the Stavanger University Hospital, was the first Norwegian hospital that initiated a pilot project to adopt the same equipment for TVC with the purpose of possibly reducing the frequency of hospital re-admissions and improving quality of life for the COPD patient.

The main aim of this study was to evaluate the effects of TVC, between the respiratory nurse in the hospital and the COPD patient at home, on the frequency of hospital re-admissions within 6 and 12 months after discharge from Stavanger University Hospital for COPD exacerbation. Our secondary aim was to assess the impact of TVC on the length of the recurrent hospital stays within 6 and 12 months follow up after TVC. Moreover, we wanted to assess patient satisfaction related to TVC.

## Methods

### Study design

The study is conducted as a retrospective uni-center observation study of a population of COPD patients who after discharge from hospital, or during outpatient treatment for acute COPD exacerbation, was monitored for 2 weeks by TVC between the specialist respiratory nurse at the hospital in Egersund, Stavanger University Hospital, and the COPD patient at home during the pilot project period from 16 April 2010 to 31 December 2011. Two weeks’ intervention was based on the benefits observed among the Danish patients who had the TVC equipment at home for about one week followed by at least one phone-call [17]. The main purpose was to teach patients to increase empowerment, self-care and correct medication in a more stabilised condition than the acute phase of hospitalisation. In order to study a possible change in amount of hospital admissions due to acute COPD exacerbations we have compared the frequencies and durations of such events before and after TVC in comparable time periods. Admissions due to other causes than acute COPD exacerbations have not been included in this study. The index hospitalisation was not included.

### Study population

All COPD patients living in the southern part of Rogaland county in Western Norway, with a habitual value of FEV_1_< 50%, who had been monitored at home by TVC following discharge after emergency hospitalisation for COPD exacerbation at Stavanger University Hospital or DMC in Egersund, or who had tele-monitoring at home under outpatient treatment for acute COPD deterioration during the pilot project period, were included in the follow up registration study. COPD exacerbation was defined as increased need for COPD medication due to worsened dyspnoea, cough or increased amount of or purulent sputum (not due to any other underlying lung or heart disease), with or without a pneumonia.

Written informed consent was obtained from each participant, and inclusion criteria comprise willingness to participate, age > 40 years, no active malignant or any other disease with prognostic life expectancy shorter than 12 months, not previously included in the study, residence outside in-service-housing with care or nursing homes, and ability to communicate. Thus, also patients with hearing impairment, aphasia or dementia were excluded from the TVC service and the study.

In-hospital treatment was in accordance with hospital policy and Norwegian National guidelines for prevention, diagnosis and monitoring of individuals with COPD and included bronchodilators inhaled by nebuliser, systemic steroids, antibiotics, oxygen supply, chest physiotherapy and when needed non-invasive ventilatory support (NIV) according to BTS guidelines [18].

Power calculations have not been performed for the purpose of this pilot study, because of too many unknown factors. The results of this study will form the basis of power calculations for a future prospective randomised study.

### Telemedicine video-consultation (TVC), patient monitoring and follow up

The telemedicine equipment consisted of a computer with a web camera with a microphone, through which the patient at home and the specially trained nurse in hospital were able to communicate, and also comprised requisites to measure the patient’s oxygen saturation and heart rate, and to perform a spirometry. The results were transferred to the hospital by a secure internet line. The computer had two buttons; one to contact the nurse according to daily appointments for TVC on week-days during day-time, and one alarm button to be pressed in case of acute need for consultation whenever necessary, 24 hours a day the whole week.

During the TVC the nurse made clinical observations according to a checklist (Table 1), measured oxygen saturation, and advised the patient how to cope with COPD related symptoms, use of medication and how to maintain activity of daily life and physical activity. The nurse could confer the patient with the doctor in the hospital, a physiotherapist or an occupational therapist, or advise the patient to consult a general practitioner or a home care nurse.

**Table 1:** Operational checklist in daily use by nurses during TVC


• Patient number
• General condition
• Consciousness
• Anxiety
• Food intake
• Sleep
• Level of functioning
• Physical activity
• Inhalation technique
• Number of short-acting inhalations last day
• Use of on-going inhalation medication
• Use of antibiotics
• Use of systemic steroids
• Coughing
• Sputum colour
• Sputum volume
• Sputum consistency
• Use of accessory breathing muscles
• Prolonged expiration
• Heart rate (beats/minute)
• Use of oxygen supply
• Oxygen saturation (%)
• Other medication
• Marks [consultations with doctor, home-nurse, physiotherapist or ergotherapist, emergency calls]

A detailed patient history comprising demographic data, social status, smoking habits, body mass index (BMI), use of medication, comorbidity and the habitual lung function were registered. Dates of admittance and discharge were noted. Retrospectively, medical records were scrutinised for re-admissions due to COPD exacerbations at 6 and 12 months follow up. Frequency of and date of re-admissions, length of hospital stay, and clinical data were recorded. Also frequency of admission due to COPD exacerbation and length of hospital stay(s) during the last 6 and 12 months prior to the TVC were recorded. Finally, all patients were encouraged to complete a questionnaire concerning patient satisfaction and impact on patient’s quality of life. All answers were registered anonymously.

Our primary endpoint was number of patients re-admitted and frequency of hospital re-admissions due to acute COPD exacerbations during 6 and 12 months follow up after TVC.

Our secondary endpoints included length of recurrent hospital stay(s). As we have no control group, our data are compared to similar data from the 6 and 12 months preceding TVC. Finally, we wanted to assess patient satisfaction related to TVC. All patients were encouraged to complete a questionnaire (Table 2) concerning patient satisfaction and impact on patient’s quality of life. The questionnaire, together with an explanation, was sent to the patient when finalizing the TVC. All answers were registered anonymously.

**Table 2:** Questions to patients regarding patient satisfaction and user friendliness of TVC equipment


**1. How do you usually feel at discharge from hospital after COPD exacerbation?**
Very safe – Safe – Unsafe – Very unsafe
**2. At discharge the TVC service made me feel**
Very safe – Safe – Unsafe – Very unsafe
**3. How do you consider the significance of the TVC for your ability to cope with your COPD related problems?**
**-** Great significance
- Some significance
- No significance
**4. Retrospectively, if you could choose, would you prefer a telephone consultation rather than tele-video-consultation?**
- I would prefer talking to the nurse by phone
- I would prefer the tele-video-consultation
**5. Which of the following statement fits you best concerning your experience of user-friendliness of the TVC equipment?**
- It was easy to operate the TVC equipment
- It was a bit difficult to operate the TVC equipment
- I could not manage to operate the TVC equipment
**6. Who was operating the tele-video conferencing system at home?**
- You, every time
- A friend or a relative operated the tele-video-conferencing system every time
- Different persons operated the tele-video-conferencing system every time
- The tele-video-conferencing system was not in use

The study was approved by the Regional Board of Research Ethics and conducted in accordance with the Declaration of Helsinki [19,20].

### Statistical methods

Continuously distributed variables of baseline characteristics were given as mean ± SEM, while variables with more skewed distributions were given as median and upper and lower quartiles.

The Shapiro-Wilk test for normality was performed to study the distribution of parameters. A chi-square test was applied to compare the frequency of re-admissions during 6 and 12 months following TVC to the frequency of hospital admissions during the last 6 or 12 months prior to TVC. The total number of days in hospital during the 6 and 12 months prior to TVC was compared to number of days in hospital during 6 and 12 months following TVC by a paired t-test. Differences in baseline characteristics between patients who were re-admitted and those who were not, were analysed by a Two-sample Student’s T-test, or in case of non-normality, by the Mann–Whitney Rank Sum test. A multiple logistic regression model with re-admission (yes/no) as the dependent variable and several baseline characteristics as potential independent predictors of re-admission was applied.

A statistically significant level of p < 0.05 was applied for all tests. All statistical analyses were performed using the statistical package of SigmaPlot Version 12.

## Results

### Baseline data

Ninety-nine patients were followed for 6 months after TVC, out of whom 56 could be followed for a total of 12 months before the closure of the study on 31 December 2012. Three patients died in an outpatient setting without being re-admitted, and only 3 patients died after 6 months follow up.

The baseline demographic and clinical characteristics of all patients with 6 months follow up are presented in Table 3. The patients were residents of 13 different municipalities in Rogaland; 44% lived in the two most densely populated municipalities. Prior to TVC, 78 patients (78.8%) were hospitalised at Stavanger University Hospital, while the remaining had outpatient treatment for the COPD exacerbation prior to TVC. During index admission, all patients inhaled ipratropium and salbutamol by nebuliser, and all except 3 patients were treated with systemic corticosteroids, while 94.9% of patients received antibiotics**.** Respiratory failure with need for oxygen supply during hospital stay was reported among 64.6% of patients, and 11.5% of patients, presenting with pH < 7.35 and hypercapnia, were treated with non-invasive ventilatory support according to BTS guidelines [18]. The high rate of 85.7% of patients inhaling combination products of long acting beta-2-agonist and corticosteroid as part of the on-going regular medication reflects the severity and the symptom burden of COPD in this population. Nearly all patients were treated with either ipratropium or tiotropium. Twenty-seven patients (27.3%) were classified in COPD Gold stadium IV, with FEV_1_ ≤ 30% of expected.

**Table 3:** Baseline demographic and clinical characteristics (n = 99)

	
Age [ys; mean (SEM)]	70.6 (0.95)
Men [n (%)]	45 (45.9)
Living alone [n (%)]	47 (47.5)
Having Home nursing [n (%)]	46 (46.5)
Current smoker [n (%)]	27 (27.3)
Ex-smoker [n (%)]	69 (69.7)
BMI [kg/m^2^; mean (SEM)]	23.4 (0.63)
Cardiovascular disease [n (%)]	51 (57.6)
Depression [n (%)]	40 (40.4)
FEV_1_ [liter; mean (SEM)]	0.99 (0.07)
FEV_1_% [%; mean (SEM)]	36.4 (1.61)
LTOT* at home [n (%)]	16 (16.2)
NIV** at home [n (%)]	15 (15.2)

LTOT*, Long Term Oxygen Treatment; NIV**, Non-Invasive Ventilatory Support.

The patients were monitored by TVC for 14.3 (0.63) days [mean (SEM)], consulting 3 (2–4) nurses [median (interquartile range)] through 10 (9–11) planned and agreed consultations [median (interquartile range)]. There were no emergency calls during this pilot phase.

During TVC 12 patients started systemic steroid therapy and 14 patients started antibiotics, out of whom 11 patients started combination therapy of systemic steroids and antibiotics. The oxygen saturation remained unchanged from TVC start (9.3.9% ± 0.29%) to end of TVC (93.8 ± 0.46), p = 0.26.

### Follow up data

#### ***Frequency of re-admission due to acute COPD exacerbations***

During 6 and 12 months follow up after TVC at home, we could not observe a statistically significant reduction in number of patients re-admitted due to COPD exacerbations, as compared to the last 6 and 12 months prior to TVC. Within 6 months follow up after TVC, 50 patients (50.5%) were re-admitted due to COPD exacerbations, and 50 patients (50.5%) had been admitted the preceding 6 months, p = 0.887. However, following each of the 50 patients hospitalised during the 6 months prior to TVC, 19 were not re-admitted within 6 months following TVC. Re-admissions due to other causes than acute COPD exacerbations were not included in this study.

Within 12 months follow up after TVC, 41 patients (73.2%) were re-admitted due to COPD-exacerbations, while 43 patients (76.8%) had been admitted the preceding 12 months, p = 0.827. Following each of the 43 patients hospitalised during the 12 months prior to TVC, 12 were not re-admitted within 12 months following TVC.

The frequency distribution of re-admissions due to COPD exacerbations as compared to admissions 6 and 12 months preceding TVC is depicted in Tables 4 and 5.

**Table 4:** Frequency distribution of admissions due to COPD exacerbations among patients followed for 6 months

**Number of hospital stays**	**0**	**1**	**≥ 2**
Re-admission (n)	49	14	36
Admission prior to TVC (n)	49	24	26

Number of patients with 0, 1 or ≥ 2 re-admissions within 6 months follow up after TVC as compared to during the last 6 months prior to TVC; n, number of patients.

**Table 5:** Frequency distribution of admissions due to COPD exacerbations among patients followed for 12 months

**Number of hospital stays**	**0**	**1**	**≥ 2**
Re-admission (n)	15	14	27
Admission prior to TVC (n)	13	16	27

Number of patients with 0, 1 or ≥ 2 re-admissions within 12 months follow up after TVC as compared to during the last 12 months prior to TVC; n, number of patients.

Patients who were re-admitted, were slightly older than those who were not, with a mean age of 72.4 (1.29) vs 68.5 (1.36) years, respectively. The re-admitted ones and those who were not re-admitted did not differ regarding to sex, baseline FEV_1_, cardiovascular comorbidity, blood gas values or low BMI (< 20). In the multiple logistic regression model age [odds ratio (OR) 1.11 (95% CI 1.01 – 1.22, p = 0.043), BMI [OR 1.13 (95% CI 1.01 – 1.27, p = 0.032] and male sex [OR 5.12 (95% CI 1.18 – 22.22, p = 0.029] were identified as predictors of re-admission, while pulmonary function parameters, a history of previous admission, current smoking or status of living alone were not associated with re-admission.

#### ***Length of hospital stays due to acute COPD exacerbations before and after TVC***

The length of re-admission hospital stays (days; mean ± SEM) due to COPD exacerbations within 12 months following TVC was significantly reduced as compared to 12 months prior to TVC, as depicted in Table 6. A similar tendency was noted for patients followed for 6 months, but did not reach statistical significance (Table 6). Assessing only patients hospitalised the last 6 and 12 months prior to TVC, we noted significantly shorter hospital stays when re-admitted within 6 and 12 months following TVC, p = 0.033 and p = 0.001, respectively (Table 7).

**Table 6:** Mean length of hospital stays before and after TVC during comparable time periods (all patients)

**Hospital stays**	**Within 12 months prior to TVC**	**Within 12 months following TVC**	**Within 6 months prior to TVC**	**Within 6 months following TVC**
**Days**	11.6	6.3^*^	7.1	4.5^**^
**[mean (SEM)]**	(2.16)	(0.80)	(1,92)	(0.64)

Mean length of hospital stays (days) before and after TVC during comparable time periods in all patients followed for 6 months (n = 99) and for 12 months (n = 56) after TVC.

^*^p = 0.023, ^**^p = 0.200 (Paired t-test).

**Table 7:** Mean length of hospital stays before and after TVC in patients admitted prior to TVC

**Hospital stays**	**Within 12 months prior to TVC**	**Within 12 months following TVC**	**Within 6 months prior to TVC**	**Within 6 months following TVC**
**Days**	15.1	6.1^*^	11.2	6.0^**^
**[mean (SEM)]**	(2.59)	(0.85)	(2.11)	(1.01)
		(n = 43)		(n = 50)

Mean length of hospital stays (days) when admitted within the last 6 months (n = 50) and 12 months (n = 43) prior to TVC as compared to length of re-admissions (days) within 6 and 12 months following TVC.

^*^p = 0.001, ** p = 0.033 (Paired t-test).

#### ***Patient satisfaction***

Among survivors, the response rate to the survey was 90%. While 52.4% of patients responded retrospectively that they generally felt safe or very safe when discharged from hospital without TVC, 90.5% of patients retrospectively reported safety when discharged to TVC at home, p < 0.001. Nearly 48% of patients found that TVC had great importance for their further management of their COPD related problems. Ninety-six percent of patients found the telemedicine equipment easy to operate, and 95.2% of patients reported that they handled the tele-video conferencing system on their own.

Moreover, 78.7% of patients preferred TVC above only phone calls.

There were few technical problems. Cancellation of the TVC occurred some times due to internal IT issues. Reproducible spirometry results were difficult to obtain in many patients, thus spirometry results are not given in our report.

## Discussion

Our study is the first to demonstrate a marked reduction in the length of hospital re-admissions in patients with COPD during a 12 month period following intervention with TVC, as compared to the last 12 months prior to TVC. Moreover, patients who had been admitted the last 6 or 12 months prior to TVC, had shorter hospital stays when re-admitted following TVC, meaning important savings in health costs for the community and improved quality of life for the patient. Only confirmation of these findings in a prospective, randomised, controlled trial could position TVC as an important new contribution to standard management of these patients.

In this retrospective pilot study we could, however, not demonstrate a reduction in the rate of hospital re-admission due to COPD exacerbations during 6 and 12 months follow up after TVC, as compared to prior to TVC. Thus, our observations are not in agreement with the Danish study using the same equipment [17], demonstrating 10% reduction in re-admissions due to COPD exacerbations in the TVC group as compared to a control group. However, the observational period after TVC in that study was 28 days.

A previous history of COPD exacerbation has been suggested to be the most reliable predictor of COPD exacerbations [5,21]. Additionally, data from the Evaluation of COPD Longitudinally to Identify Predictive Surrogate Endpoints (ECLIPSE) [5] showed that 33% with COPD stage 3 and 47% with stage 4 defined according to the Global Initiative for Chronic Obstructive Lung Disease (GOLD) [22] had ≥ 2 exacerbations in the first year of follow up. Our patients are characterised by COPD stage 3–4, with a mean FEV_1_ of 36.3%, and 67.9% had been admitted the last year. In accordance with previous observations [17,23], higher age and male gender was associated with an increased risk of re-admittance in our study.

Our main finding was the reduction in days per admission when in need of hospitalisation. Several factors could have influenced this observation. In addition to the introduction of TVC, a change in treatment or hospitalisation policies might have influenced this. There are, however, no new treatments introduced in the study period at the hospital. In addition, on-going regular medication was similar at inclusion and at discharge from index hospital stay (prior to TVC), and no change in conventional medical therapy could explain this difference. Regarding hospitalisation policies, “The new Coordination Reform”, guaranteeing immediate help from the municipality health care system when discharged from hospital, such as a place in a nursing home, came into force in Norway the 1^st^ of January 2012. This reform does not seem to explain the shorter length of hospital stays in this study, as the patients receiving TVC in this study, were not candidates for nursing homes. Besides, more than 60% of the re-admissions occurred among the 49 first included patients whose 12 months follow up were completed before “The Coordination Reform” came into force.

We therefore believe that the observed changes in length of hospital stays were related to the introduction of the TVC. Many advances related to the TVC could contribute to the benefit of reduced length of hospital stays when re-admitted. During the TVC period, the specially trained nurse not only daily monitored and advised each patient according to personal needs to improve the actual health condition. Also, great effort was invested in advising and teaching each patient to increase the empowerment and competence for good self-care, concerning correct medication use, inhalation technique, appropriate physical activity, pulmonary drainage, dealing with stress and anxiety to prevent and to cope with future exacerbations. However, our study has not been designed to directly evaluate the influence of TVC on each of those factors.

An interdisciplinary team was available for consultation when needed, including among others a physiotherapist and an occupational therapist, who could for example easily facilitate home conditions, and thereby also enable a faster and smoother future hospital discharge. The discharge policy of the hospital, defined in the hospital’s quality criteria, has not changed before and after the TVC during the study period, ensuring a stabilised patient without need for fast-acting bronchodilators more frequently than every 4 hour, whose ability to eat and sleep is not limited by breathlessness, ensuring the patient appropriate home conditions, and that the patient, relatives and eventually nursing staff can administer the prescribed treatment, that an ambulatory patient can walk at least 20 meters, and the need of interdisciplinary rehabilitation should have been considered, all in order to guarantee that the patient will manage at home. The marked reduction after TVC of the relatively long hospital stay might therefore be related to a facilitated discharge process and safeguarding of home conditions offered by TVC. However, home nursing was equally frequent among those who were re-admitted and those who were not. Thus, home-nursing does not seem to prevent re-admissions. There was no change in the hospital’s discharge policy during the study period.

The self-perceived patient satisfaction and the increased coping skills following TVC are also of major importance for the feeling of safety at home, which was reported high in the enquiry.

The marked reduction in use of hospital resources after introduction of TVC in this pilot study gives promise for a better management of COPD patients. This could induce both a better life and feeling of safety for the patients and reduce the costs for the society. Further, the results presented in this report, are based on the experience from the early start of TVC at our site with a strict use of the equipment for 2 weeks. The accumulated experience through the development of the program indicates even better effects by applying an individualised approach with some patients with shorter follow up and longer in others, based on their individual condition and needs. It is therefore possible that a future, more flexible, individually adapted duration of TVC monitoring of patients might even contribute to increase periods free of re-admissions, rather than the strict, pre-determined period of 14 days of TVC monitoring, used in our study, irrespective of patient condition and needs.

For majority of patients the need of re-admission has been decided by occurrence of an infective exacerbation, as nearly 70% of patients re-admitted, required antibiotic therapy during the first re-admission. Thus, these re-admissions are hard to avoid, but the length of re-admission hospital stays were shortened, as already discussed.

The major limitation of this study is, of course, the retrospective design and the lack of a control group. This pilot study is, however, inspiring to perform a randomised, controlled trial evaluating the effect of TVC on re-admissions and length of hospital stays due to COPD exacerbations. Moreover, DMC was the first centre in Norway applying TVC, being a pioneer, establishing a new therapeutic approach in our region, and the concept has probably improved by time, gaining more experience. Including also the first patients participating might have influenced the results of our investigation. On the other hand, the TVC system has been user friendly both for the patients and the nurses, experiencing few technical problems, the nurses have been well skilled and trained, and both the patient confidence in the TVC service and the patient satisfaction were high.

Cost-benefit analysis has never been an aim of this pilot study, and future research is needed to confirm the cost effectiveness of TVC for COPD patients.

## Conclusions

In conclusion, despite the failure to demonstrate a reduced frequency of re-admittance within 6 and 12 months following TVC, as compared to pre-TVC in this pilot study, the length of the re-admission stays within 12 months following TVC was considerably reduced as compared to pre-TVC. Patients who had been admitted prior to TVC, had shorter hospital stays when re-admitted post-TVC. The patient satisfaction was high. Future randomised, controlled trials must be performed to confirm the results of this pilot study and to verify that the TVC does not offer a lower solution to hospital safety.

## Abbreviations

BMI: Body mass index; COPD: Chronic obstructive pulmonary disease; DMC: Dalane District Medical Centre; FEV1: Forced expiratory volume in first second; HR: Hazard ratio; LTOT: Long term oxygen treatment; NIV: Non-invasive ventilatory support; SEM: Standard error of the mean; TVC: Telemedicine video-consultation.

## Competing interests

The authors declare that they have no competing interests.

## Authors’ contributions

SS participated in the design of the study, made contributions to interpretation of data and drafted the article. JPL made substantial contributions to the design of the study, interpretation of data and revision of the manuscript. JBA participated in the design and coordination of the study, collection of and interpretation of data. HG participated in the design of the study, performed the statistical analyses, and made contributions to interpretation of data and helped to draft the manuscript. All authors read and approved the final manuscript.
